# What If Not All Metabolites from the Uremic Toxin Generating Pathways Are Toxic? A Hypothesis

**DOI:** 10.3390/toxins14030221

**Published:** 2022-03-17

**Authors:** Raymond Vanholder, Sanjay K. Nigam, Stéphane Burtey, Griet Glorieux

**Affiliations:** 1 Nephrology Section, Department of Internal Medicine and Pediatrics, Ghent University Hospital, 9000 Ghent, Belgium; griet.glorieux@ugent.be; 2 Departments of Pediatrics and Medicine (Nephrology), University of California San Diego, La Jolla, CA 92093, USA; snigam@health.ucsd.edu; 3 Centre de Recherche en Cardiovasculaire et Nutrition (C2VN), Institut de la Santé et de la Recherche Médicale (INSERM), Institut National de la Recherche pour l’Agriculture, l’Alimentation et l’Environnement (INRAE), Aix Marseille University, 13005 Marseille, France; stephane.burtey@univ-amu.fr

**Keywords:** uremia, uremic toxins, kidney disease, tryptophan, metabolism, indoles, kynurenines, aryl hydrocarbon receptor, remote sensing and signaling theory, gut microbiome

## Abstract

The topic of uremic toxicity has received broad attention from the nephrological community over the past few decades. An aspect that is much less often considered is the possibility that the metabolic pathways that generate uremic toxins also may produce molecules that benefit body functions. Here, we discuss this dualism based on the example of tryptophan-derived metabolites, which comprise elements that are mainly toxic, such as indoxyl sulfate, kynurenine and kynurenic acid, but also beneficial compounds, such as indole, melatonin and indole-3-propionic acid, and ambivalent (beneficial for some aspects and harmful for others) compounds such as serotonin. This dualism can also be perceived at the level of the main receptor of the tryptophan-derived metabolites, the aryl hydrocarbon receptor (AHR), which has also been linked to both harm and benefit. We hypothesize that these beneficial effects are the reason why uremic toxin generation remained preserved throughout evolution. This duality is also not unique for the tryptophan-derived metabolites, and in this broader context we discuss the remote sensing and signaling theory (RSST). The RSST proposes that transporters (e.g., organic anion transporter 1—OAT1; ATP-binding cassette transporter G—ABCG2) and drug metabolizing enzymes form a large network of proteins interacting to promote small molecule remote communication at the inter-organ (e.g., gut–liver–heart–brain–kidney) and inter-organismal (e.g., gut microbe–host) levels. These small molecules include gut microbe-derived uremic toxins as well as beneficial molecules such as those discussed here. We emphasize that this positive side of uremic metabolite production needs more attention, and that this dualism especially needs to be considered when assessing and conceiving of therapeutic interventions. These homeostatic considerations are central to the RSST and suggest that interventions be aimed at preserving or restoring the balance between positive and negative components rather than eliminating them all without distinction.

## 1. Introduction

The expected remaining lifetime for people with advanced chronic kidney disease (CKD) is more than halved across all age strata [1]. The average five-year survival of patients starting dialysis is lower than that of cancer, upon diagnosis [2]. The majority of CKD patients will die, mostly from cardio-vascular complications, before they reach the stage of dialysis or transplantation [3]. The calculated annual health care cost in Europe for CKD is higher than that of cancer or diabetes [2]. Acute kidney injury (AKI) only adds to outcome burden and cost [4,5].

This dismal picture can, to a considerable extent, be attributed to the accumulation of toxic metabolites (uremic toxins) that are eliminated by healthy kidneys via the urine [6]. These uremic toxins have been related to many of the lethal complications of kidney disease, especially cardio-vascular and infectious diseases and the progression of kidney insufficiency [6], but also to a number of distressing patient-related outcomes, such as cognitive dysfunction or itching [7,8], which are not fatal but affect quality of life substantially [2]. The pathophysiologic mechanisms defining uremic syndrome have received ample attention from the nephrologic community over the past few decades [6,9].

However, each shadow has a bright side. This duality exists throughout biology. Bacteria and yeasts cause killer diseases but are also used for the production of relatively benign foods, drinks or ingredients such as kombucha, yoghurt, soybean sauce and cheese. Duality has in antiquity been symbolized by the door deity Janus, who marked the separation between the inside and the outside of the house.

**Figure 1:**
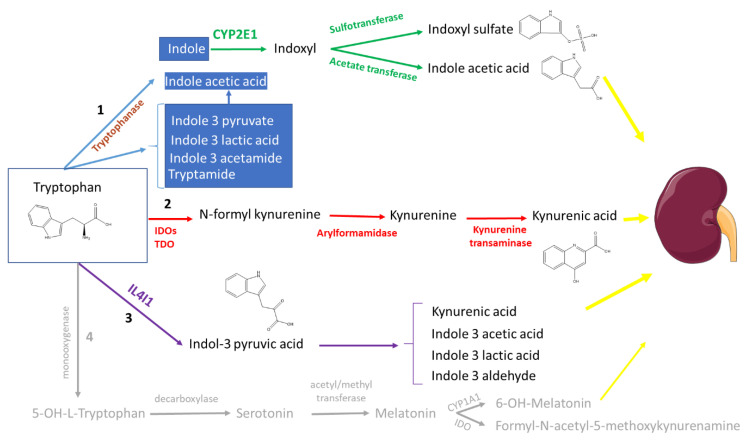
Global metabolic pathways of tryptophan according to the most recent insights. Different enzymes are involved in the generation of uremic toxins and activators of aryl hydrocarbon receptor (AhR). (1) Tryptophanase; (2) indoleamine-2,3-dioxygenases (IDOs) and tryptophan-2,3-dioxgenase (TDO) and (3) the newly identified interleukin 4-induced-1 (IL4I1). Indole is further metabolized in the liver (green arrows) by cytochrome P450 family 2 subfamily E member 1 (CYP2E1), resulting in indoxyl and indoxyl sulfate (sulfotransferase) and indole-3-acetic acid (acetate transferase). Both the IDOs/TDO (red arrows) and IL4I1-dependent pathways (purple arrows) are involved in the generation of kynurenic acid. The end-metabolites are excreted by the kidneys (yellow arrows). For the sake of completeness, in grey, (4) the serotonin pathway.

As we repetitively reviewed and ranked solutes for their toxicity [6,10,11], we realized that some metabolically related compounds were not toxic and even beneficial. However, the question as to how far the biologic ambiguity mentioned above also exists for uremic toxin metabolism has rarely been addressed or reviewed in depth: what if our tunnel vision disregards related compounds with beneficial impacts or if the so-called toxins are not unequivocally toxic? This question may be critical if successful efforts to reduce toxin concentrations have as a downside diminishing the benefits. We will consider this dilemma based on a detailed analysis of the functional effects of the metabolic cascade of tryptophan, including consideration of the recently described interleukin 4-induced-1 (IL4I1) pathway [12] (Figure 1). After having reviewed the functional properties of the main tryptophan metabolites, we will consider why the generation of potentially lethal molecules such as indoxyl sulfate, kynurenine, or indole-3-acetic acid [13,14,15] has not been switched off by natural selection or reproductive isolation.

Our journey will lead us to consider biochemistry, (patho-)physiology, evolutionary biology, history, lifestyle and genetics, in order to propose a comprehensive hypothesis on solute metabolism in CKD. This will also lead to the consideration of the remote sensing and signaling theory (RSST) of small molecule inter-organ and inter-organismal communication mediated by drug transporters (e.g., organic anion transporter 1—OAT1; ATP-binding cassette transporter G—ABCG2) and drug metabolizing enzymes [16,17]. However, the field of uremic toxicity is rapidly advancing and encompasses many disciplines, and the viewpoint presented here might need to be fine-tuned in the future as novel information and interpretations emerge.

## 2. Tryptophan Metabolites: The Good, the Bad, and the Ambivalent

**Figure 2:**
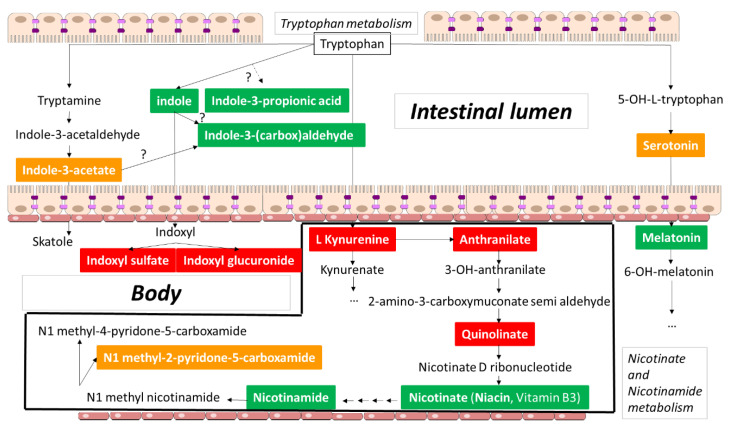
Main metabolites of tryptophan. Extracted from Kyoto Encyclopedia of Genes and Genomes) (KEGG) pathways). The compounds with a colored background are discussed in the paper. Green background: mainly positive effect; red background: mainly negative effect; orange background: ambiguous data. Black frame: kynurenic acid pathway. Upper part: intestinal lumen; lower part: inside the body. Indole-3 propionic acid and Indole-3-(carbox)aldehyde are not mentioned in the KEGG pathways.

In what follows we will, based on the collected information, tentatively subdivide tryptophan metabolites (Table 1) into mainly toxic, mainly beneficial, and essentially ambivalent (or mixed) molecules (Figure 2). We make these categorizations with the understanding that simple subdivisions may need to be reconsidered; for instance, even those molecules we currently refer to as “toxic” are increasingly believed to also play roles in signaling through their influence upon nuclear receptors, G-protein coupled receptors (GPCRs) and kinases. After discussing selected molecules below, we provide a conceptual framework for a more nuanced interpretation of the roles of these molecules in local and systemic homeostasis and pathophysiology: the RSST. We then turn to evolutionary concerns, asking the question of why uremic toxins have persisted throughout evolution.

### 2.1. The (Mainly) Toxic Molecules

#### 2.1.1. Indoxyl Sulfate

Few uremic retention solutes have received as much attention as indoxyl sulfate. Hundreds of studies have pointed to its effects contributing to uremic syndrome [6]. Although the correctness of the concentrations analyzed has been questioned for some studies [18,19], a considerable number of high-quality studies underscore its multifunctional burden [13], including its roles in cardio-vascular disease, kidney and heart fibrosis, thrombogenicity, metabolic and hormonal dysfunction, inflammation and chronic kidney disease-mineral and bone disorder (CKD-MBD) [6]. More recently reported functional defects include neurotoxicity [20,21,22], intestinal epithelial [23] and hematologic alterations [24,25,26], sarcopenia [27], loss of muscle mass [28], disturbed drug removal [29], and accelerated cell senescence [30]. Undeniably, indoxyl sulfate is responsible for a plethora of negative effects, and it is ranked among the most important uremic toxins [6]. However, these generally negative effects can be supportive in specific conditions such as breast cancer, in which tryptophan metabolism is suppressed. Supplementation of indoxyl sulfate to restore concentrations to the normal reference range exerted cytostatic properties via a reduction in cell proliferation and the induction of oxidative and nitrosative stress [31].

#### 2.1.2. Indoxyl Glucuronide

Indoxyl glucuronide has rarely been studied. One study showed inhibition of hypoxia-inducible factor (HIF)-dependent erythropoietin expression [32]. Indoxyl glucuronide is also one of the uremic solutes inhibiting organic cation transporter-2 (OCT-2), which plays a role in the kidney excretion of drugs and environmental toxins [33].

#### 2.1.3. Kynurenine and Kynurenic Acid

The kynurenine pathway follows a separate metabolic route from that of the indole derivatives (Figure 1 and Figure 2) [34]. This axis is mainly active in the liver but, especially after immune activation, also in other tissues [34]. Biologic studies essentially focused on kynurenine and kynurenic acid.

Kynurenine and/or kynurenic acid have been linked to cardio-vascular disease, inflammation, thrombogenicity, metabolic dysfunction and neurotoxicity [6]. Kynurenic acid has especially been studied as a regulator of neurologic function, whereby it acts as an N-methyl-D-aspartate (NMDA) receptor antagonist and functionally equilibrates with quinolinic acid, an NMDA receptor agonist [34]. A targeted metabolomic study in CKD found a significant relationship between kynurenine concentration and thrombotic events [14]. Other studies reported an impact on bone quality and strength [35,36]. Thus, kynurenine and kynurenic acid have a large array of negative effects, and have also been ranked among the most important uremic toxins [6].

#### 2.1.4. Anthranilic Acid

Other metabolites of the kynurenic pathway have received less attention. One of these, anthranilic acid, has shown a positive correlation with fibrinolytic parameters in early CKD, while this relationship was inverted in more advanced stages [37]. Anthranilic acid is, at different stages of CKD, associated with markers of endothelial dysfunction [38]. However, direct causation has not been proven.

#### 2.1.5. Quinolinic Acid

Quinolinic acid is especially known as a brain excitotoxin [39], but has also been linked to inflammation and inhibition of erythropoiesis [6], and more recently to a species-dependent effect on hemostasis, being prothrombotic in mice but the opposite in rats [40]. As with anthranilic acid (see above), in CKD, quinolinic acid is also associated with markers of endothelial dysfunction [38]. In addition, quinolinic acid is also related to intima-media thickness [38].

### 2.2. The (Mainly) Beneficial Molecules

#### 2.2.1. Tryptophan

Tryptophan cannot be generated by the body and is thus an essential amino acid. There is limited evidence of an antidepressive effect of tryptophan-containing food supplements [41], although excessive intake has been linked to potentially lethal toxicity (eosinophilia-myalgia syndrome). Whether this complication is attributable to tryptophan as such or to a contaminant metabolite remains a matter of debate [41,42]. A diet rich in L-tryptophan preserved the secretion of insulin and delayed the progression of hereditary type 2 diabetes in rats [43]. It may, however, be questioned whether this effect should be attributed to tryptophan per se or to its downstream metabolites.

#### 2.2.2. Indole

Indole is the mother compound of a large array of metabolites, which are generated in several organ systems, but mainly in the liver. Studies of intestinal indole generation observed no changes in generation at all stages of CKD, including kidney failure treated by dialysis [44]. Indole is known as a promotor of intestinal wall integrity and repair [45,46,47], and acts as an anti-inflammatory agent in enteropathy induced by non-steroidal anti-inflammatory drugs (NSAID) [47] and by counteracting the detrimental effects of lipopolysaccharides in the liver [48]. Indole also attenuates the virulence of several pathogens, such as *Candida albicans*, *Staphyloccus aureus* and *Salmonella* [49,50].

Alongside these positive effects, one single study reported portal hypertension, be it after supranormal doses [51], and in another study, indole administration to rats was associated with faster progression of kidney dysfunction and glomerular sclerosis [52]. As these two studies are isolated reports compared to a broad consensus on the advantages, we categorized indole as a beneficial compound. In addition, here also it can be questioned whether the responsible compound is indole or one of its downstream metabolites.

#### 2.2.3. Indole-3-propionic Acid

Indole-3-propionic acid concentration in the serum is decreased in CKD [53]. This compound has mainly been labelled as neuroprotective [54,55], with stronger anti-oxidative capacities than melatonin, and the potential to counteract Alzheimer’s [54]. Furthermore, indole-3-propionic acid attenuates steatohepatitis [56], restrains the progression of cancer [57], improves the intestinal epithelial barrier [56,58], regulates endothelial function [59], and has been associated with a lower risk of type 2 diabetes [60]. Indole-3-propionic acid reduced weight gain in rats [61], but did not protect against the cardio-metabolic effects of Western diet in mice [62]. Indole-3-propionic acid was also identified as a modulator of cardiomyocyte mitochondrial function and overall cardiac function; however, it also showed an acute benefit but a negative chronic effect, thus exemplifying in this case setting-specific dualism [63].

#### 2.2.4. Indole-3-(carbox)aldehyde

Indole-3-aldehyde is best known for its ability to promote gut epithelial barrier functions [64], but it also alters immune function, e.g., by the expression of interleukin-22, helping to stabilize gut mucosa immune homeostasis and intestinal microbial content [65]. It also modulates inflammatory injury induced by respiratory syncytial virus (RSV) in vitro [66], and displays antimicrobial activities, e.g., against both non methicillin-resistant and methicillin-resistant *Staphylococcus aureus*, and *Candida* [67]. Finally, studies in a murine model suggest protection against the metabolic syndrome [68].

#### 2.2.5. Melatonin

Melatonin is essentially secreted at night by the pineal gland and controls the sleep–wake rhythm [69]. Several systematic reviews pointed to a positive effect of exogenous melatonin on sleep disturbances [70,71,72], although administration to healthy volunteers late in the evening when natural melatonin secretion was at its maximum had no impact, in contrast to administration early in the evening [73]. Additional attributed effects include immunoregulation [74,75] and analgesia [76]. Melatonin improved mitochondrial function, glycolytic metabolism and proliferation of mesenchymal stromal and stem cells collected in mice with CKD [77]. In a murine model of acute kidney injury, melatonin inhibited transition to chronic kidney disease [78]. In a randomized controlled cross-over study in hemodialysis patients, melatonin had an immunoregulatory and an anti-inflammatory effect [79].

#### 2.2.6. Nicotinic Acid and Nicotinamide

Nicotinic acid (also referred to as niacin, although the term niacin is also used for both nicotinic acid and nicotinamide together) is generated downstream in the kynurenic pathway. About 60 mg of tryptophan is needed to generate 1 mg of nicotinic acid [80]. Also catalogued as vitamin B_3_, a niacin deficit has historically been linked to pellagra, a currently exceptional dermatologic disease, also characterized by diarrhea and dementia, that mainly occurs in impoverished populations with deficient meat consumption living on maize [81]. However, nicotinic acid and nicotinamide more recently gained new momentum as lipid metabolism regulators [82], although most randomized controlled trials could not demonstrate their benefit on hard outcomes [83,84]. Nicotinamide acts against endothelial oxidative stress, which links it to an anti-inflammatory and vasculoprotective effect [85]. In mice, nicotinamide supplementation prevented CKD progression by reducing kidney inflammation and fibrosis [86]. It also protected against acute kidney proximal tubule damage induced by metabolic acidosis [87] and against ischemic AKI [88]. In hemodialysis patients, nicotinic acid and nicotinamide have also been attributed a phosphate lowering effect [89,90]. However, one of the metabolites, 1-methyl-2-pyridone-5-carboxamide (2PY) [80,91], is dramatically increased in dialysis patients supplemented with these compounds, and has been linked to a number of both positive and negative effects [92] (see below).

### 2.3. The Ambivalent Molecules

#### 2.3.1. Serotonin

Serotonin is primarily known as a neurotransmitter, low brain levels of which are linked to poor memory and depression [93]. However, most serotonin is found outside the central nervous system. Experimental data suggest that intestinally generated serotonin impacts central nervous serotonin levels and behavior [94]. Furthermore, serotonin also functions as a regulator of cardiovascular function, bowel motility, ejaculation, and bladder control [95]. However, it also has a pro-coagulant effect [96] and is linked to itching [97,98]. Circulating serotonin is increased in CKD patients and is dramatically high during dialysis [99,100]. In an experimental CKD study, serotonin was indirectly linked to loss of bone quality [100], and, in two clinical studies successfully administering a serotonin receptor antagonist, to the relief of itching [101,102]. However, the latter two studies were not placebo controlled and the used drug also neutralized histamine, making it impossible to attribute the drug effect to serotonin antagonism [101,102].

#### 2.3.2. Indole-3-acetic Acid

Indole-3-acetic acid is directly produced by the intestinal microbiome. It has been linked to inflammation, cardiovascular disease, thrombogenicity, fibrosis and metabolic dysfunction [6], and more recently cognitive dysfunction [103]. On the other hand, indole-3-acetic acid has also been positively associated with the activation of stem cell factor (SCF), which plays a role in tissue repair, hematopoiesis and cell proliferation [104] and with anti-inflammatory and anti-oxidant activity on lipopolysaccharide stimulated macrophages [105], albeit at concentrations exceeding more than 30 times those observed in uremia. Indole-3-acetic acid is decreased in the feces of patients with inflammatory bowel disease and in mouse models of inflammatory bowel disease [106]. Inoculation of affected mice with microbiota generating tryptophan metabolites including indole-3-acetic acid improved inflammatory bowel disease in these mouse models [106]. Administration of indole-3-acetic acid to mice also attenuated non-alcoholic fatty liver induced by high-fat diet [107].

#### 2.3.3. 1-Methyl-2-pyridone-5-carboxamide

1-Methyl-2-pyridone-5-carboxamide at uremic concentrations has been linked to genomic instability and anemia [92,108]. Another study, however, pointed to a protective effect against endothelial oxidative stress [85].

### 2.4. Remote Sensing and Signaling Theory (RSST) and Its Relation to Uremia

**Figure 3:**
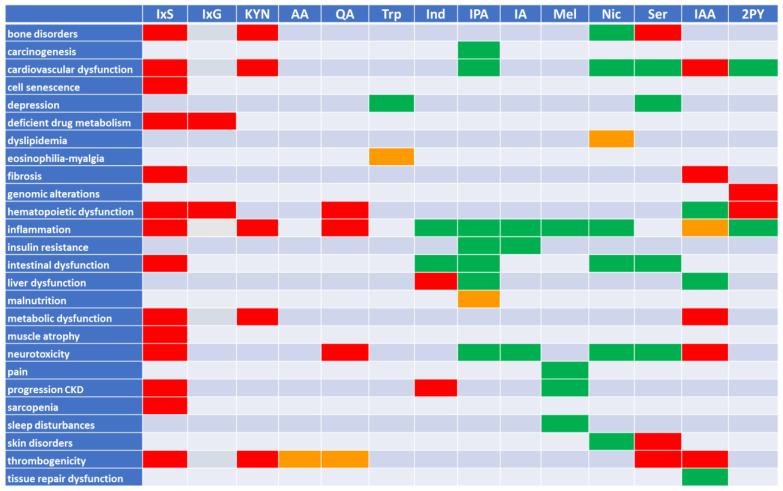
Summary of the biological effects of the tryptophan metabolites discussed in this publication. Red: negative effect; green: positive effect; orange: conflicting data. CKD: chronic kidney disease. IxS: indoxyl sulfate; IxG: indoxyl glucuronide; KYN: kynurenine/kynurenic acid; AA: anthranilic acid; QA: quinolinic acid; Trp: tryptophan; Ind: indole; IPA: indole-3-propionic acid; IA: indole-3-(carbox)aldehyde; Mel: melatonin; Nic: nicotinic acid/nicotinamide; Ser: serotonin; IAA: indole-3-acetic acid; 2PY: 1-methyl-2-pyridone-5-carboxamide.

As discussed above, uremic molecules can be largely or somewhat toxic (especially in the setting of kidney dysfunction), largely or somewhat beneficial, or both (Figure 3). We have emphasized the theme of “balance” in normal local and/or systemic physiology, or in helping to restore the system after injury—in other words: homeostasis. Many tryptophan-derived molecules, often originating from gut microbes and labeled uremic solutes or toxins, play key roles in metabolism, redox state, and signaling, and act upon nuclear receptors (transcription factors), G-protein coupled receptors, kinases, or directly affect key metabolic pathways.

**Figure 4:**
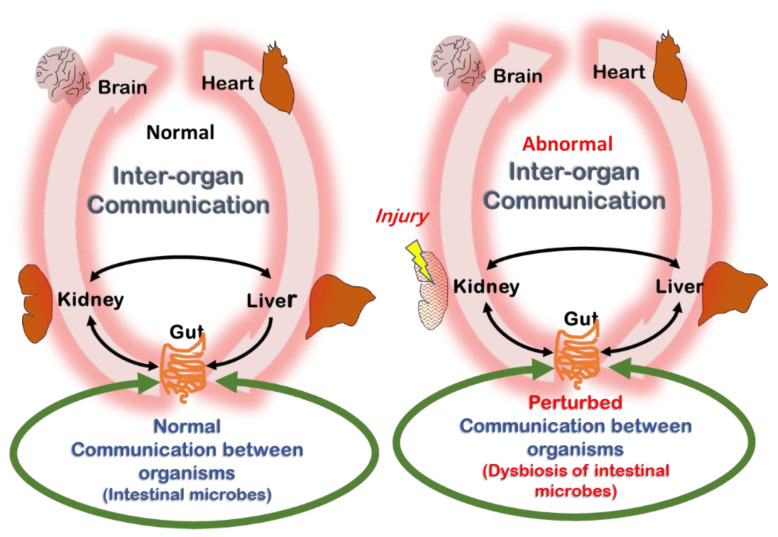
Normal and abnormal (uremic) remote sensing and signaling. Please see text for a detailed explanation of the remote sensing and signaling theory of interorgan and inter-organismal (gut microbe–host) communication via small molecules that regulate metabolism, signaling, and oxidative state. The proteins mediating these effects of small molecules include transporters, drug metabolizing enzymes, and nuclear receptors. Some of the regulated molecules include gut microbe-derived uremic toxins.

Nigam and coworkers proposed that about 600 transporters (e.g., organic anion transporters such as OAT1), “drug” metabolizing enzymes (e.g., cytochrome P450s—CYPs) and nuclear receptors (e.g., aryl hydrocarbon receptor—AHR (see below)) collaborate across organs and organisms (gut microbes–host) in a “remote sensing and signaling network” to maintain small molecule homeostasis (Figure 4) [16,17,109]. In this way, transporters, drug metabolizing enzymes (DMEs) and nuclear receptors regulate metabolic pathways, signaling, and oxidative state within and between cells, tissues, organs and organisms (e.g., gut microbes–host). Although the focus in this article as well as in other reviews on the RSST as applied to CKD [17,110] is on understanding the central role of transporters, DMEs and nuclear receptors in uremic metabolism, it is to be emphasized that the RSS theory is a general theory of small molecule communication between cells, tissues, organs, and organisms (Figure 4) [16].

This transporter and DME-based small molecule remote communication system operates in parallel with, and supports, the neuroendocrine and other classic homeostatic systems. For example, thyroid hormones, sex steroids, bioactive lipids, tryptophan derivatives, and bile acids are key players in multiple homeostatic systems, including the RSS [16,111].

Many genes in the remote sensing and signaling network seem to have been conserved throughout evolution [112]. A large number of these genes or their homologs can be found in fish and flies. Many have been shown in model organisms to subserve key functions [113]. Most of these genes are highly expressed in ducts and tubules in organs throughout the body, or at other interfaces with body fluids.

If ducts or tubules fail in one organ, the ducts and tubules of other organs try to restore the system. This occurs through the regulation of genes in the remote sensing and signaling system (e.g., transporters, DMEs) in an attempt to bring gut microbe products, uremic toxins, bile acids, fatty acids, signaling molecules, urate, energy metabolites, and other molecules back into some kind of balance/compromise [16,17].

OAT1 (solute carrier 22A6—SLC22A6) and OAT3 (SLC22A8), multi-specific members of the SLC22 transporter family, are well-known for their role in kidney drug elimination [114]. They also provide examples of how the remote sensing and signaling system operates. OAT1 and OAT3 are not only the key proximal tubule transporters involved in kidney elimination of gut microbe-derived uremic toxins; they are also central to the kidney “remote sensing” of tryptophan-derivatives, which play important roles in local and systemic metabolism, signaling and redox state regulation [115,116,117].

In the case of indoxyl sulfate, there appears to be a pathway from gut microbes to liver DMEs to RSS in proximal tubule cells in the service of maintaining homeostasis via effects on metabolism, redox state and signaling events [17,118]. This suggests how uremic compounds, while potentially toxic at high concentrations in the setting of kidney disease, may otherwise subserve important normal physiological functions [14,110,111,112,113].

Declining kidney function is linked to a disturbance of the homeostatic balance maintained by the remote sensing and signaling system (Figure 4). Nevertheless, in the setting of kidney disease, an interesting example of a homeostatic correction by RSS has also been uncovered. As kidney function declines, intestinal ABCG2, an ABC transporter which extrudes urate and possibly indoxyl sulfate into the gut, becomes more active [119]. It has been suggested that this increased intestinal activity of the ATP-binding cassette G2—ABCG2 transporter is mediated by urate and/or indoxyl sulfate [120,121]. Thus, the kidney with declining function, unable to properly eliminate urate and indoxyl sulfate due in part to the loss of OAT function, may remotely signal the intestine to eliminate them via the ABCG2 transporter, to help restore homeostasis. This example of inter-organ communication in the face of injury is unlikely to be an isolated one, and the field awaits detailed studies of how transporters and DMEs in different organs help to stabilize multi-organ physiology in the face of kidney injury—and the resulting effect on uremic solutes and toxins [122]. Multi-omics analysis in humans and animal models will likely provide important clues.

### 2.5. Summary

Tryptophan has a substantial number of metabolites, some mainly toxic, some mainly beneficial (although to our knowledge not unequivocally supported by controlled studies), and some playing an ambivalent role. This is reviewed concisely in Figure 3, which allows to see at a glance that there are as many harmful (red background) as beneficial (green background) effects, while several molecules harbor both positive and negative effects. Apart from thrombogenicity, which is negatively impacted by most involved solutes, and insulin resistance, depression and intestinal alterations that are in the majority of cases positively influenced, most effects seem to be modified to more or less equivalent degrees in both directions (Figure 3). However, even some notorious pathogens, such as kynurenine, still may play a physiological role, e.g., by outbalancing quinolinic acid, whereas niacin at the end of the essentially negative kynurenic metabolic chain has a largely positive impact. In addition, several of the tryptophan metabolites exert their activities through transport via OAT1 and activation of the multifunctional AHR. Although one should be cautious in extrapolating animal data to humans [123], it is generally accepted that the AHR not only activates the thrombogenic tissue factor [124,125,126], but also is both a positive and negative immunomodulator [127,128], and has likely regulated the detoxification of xenobiotics since long before the appearance of humans [127,129,130] (see below).

Thus, the harms and benefits of the solutes generated by the tryptophan metabolic chain cannot be unequivocally classified as negative or positive. However, whereas the shadow side of this group of molecules has mainly drawn the attention of nephrology researchers and clinicians because of its involvement in uremic toxicity, the benefits have unfortunately often been overlooked. The RSST provides something of a remedial framework, as it considers the physiological impact of remote inter-organ (e.g., gut-liver-heart-brain-kidney) and inter-organismal (microbe-host) communication via transported small molecules, including those labeled as uremic toxins [16,17]. While the theory accepts the potential toxic nature of protein-bound uremic toxins handled by proximal tubule transporters such as OAT1 and OAT3, the RSST emphasizes their roles in the regulation of metabolism, signaling and redox events in normal and pathophysiological settings [17,109].

## 3. The Dualism of the Aryl Hydrocarbon Receptor

Aryl hydrocarbon receptor was first described as the dioxin receptor in the 1970s [131]. Since then, several ligands activating this transcription factor have been described [132]. AHR is an 848-amino acid protein with a nuclear localization sequence (NLS), a DNA-binding domain close to a basic helix-loop-helix (bHLH) domain, and two domains, per-Arnt-sim (PAS) A and B. PAS A plays an important role in AHR dimerization and PAS B functions as a ligand binder [133]. Finally, there is a Q-rich domain that plays a role in the activation of transcription. After ligand binding in the cytoplasm, AHR is released from its stabilizing complex (AHR-interacting protein (AIP), cellular-sarc (c-Src), P23 and heat shock protein (HSP90)) into the nucleus and heterodimerize with AHR nuclear translocator (ARNT) [134]. This heterodimer will bind to xenobiotic response element (XRE) regulatory sequences in the promoters of many genes, mainly molecules involved in detoxification such as cytochrome P450 family 1 subfamily A member 1 (CYP1A1), CYP1A2, and CYP1B1 [135]. AHR also has a ubiquitin ligase (E3), which may play a role in the negative feedback of AHR signaling [136]. In addition to the activation of the traditional genomic pathway, AHR can play the role of a signaling molecule and activate a so-called inflammatory or non-genomic pathway that will lead to the activation of the nuclear factor-kappa-light-chain enhancer of activated B cells (NF-kB) pathway [137].

From an evolutionary point of view, AHR is one of the rare genes with a variation in the coding phase between homo sapiens (Val381) and homo neanderthalensis (Ala381), with the sapiens variant responding less well to activation by xenobiotics, an effect proposed to protect modern humans from a too strong activation of AHR by environmental pollutants such as smoke [138]. However, this hypothesis has been contradicted by recent data [139].

Activation of AHR by dioxin is associated with multiple complications. These can be either acute complications, such as chloracne [140], or long-term complications, such as lymphoma and malformative syndrome [141]. The long-term effects of AHR activation by dioxin are well known from the massive use of the herbicide Agent Orange during the Vietnam War [141]. The activation by dioxin is peculiar, as it is not degraded by the organism and leads, contrary to other agonists, to a permanent activation of AHR which is not eliminated by the physiological response. Additionally, activation of AHR by benzo(a)pyrene (BaP) has deleterious effects because it promotes the formation of oncogenic adducts by activating the expression of CYP450 [135]. During chronic kidney disease, AHR is activated and plays an important role in the pro-thrombotic [142], vasculotoxic [143] and neurotoxic [21] activities of tryptophan-derived uremic toxins [123]. AHR also induces or aggravates autoimmune diseases [144] and cancer [135], and has more recently been implicated in infectious diseases affecting the central nervous system, such as Zika [145]. Inhibition of AHR could be a key to the management of many diseases [146].

Besides these negative aspects of AHR activation, mice lacking activity of this transcription factor show multiple phenotypic alterations, confirming its important physiological role outside the response to xenobiotics. Rodents that have lost AHR show vascular abnormalities, persistence of ductus venosus, a tendency towards hypertension, hepatic abnormalities, fibrosis, small liver abnormalities and immune system abnormalities mainly associated with the mucosa [147]. Animal models confirmed the necessary role of AHR in physiology. Loss of AHR studies in multiple species ranging from Caenorhabditis elegans to Drosophila and mice show that the loss of AHR elicits a decrease in healthy life expectancy [148]. A lack of activation reduces the physical capacity of these animals with age, whereas supplementation with indole has a beneficial effect by increasing healthy life expectancy. A defect of AHR activation plays an important role in ageing [149]. Hence, activation of AHR during CKD could protect against the complications related to uremic syndrome. Furthermore, it has been shown that AHR plays a crucial role in keeping the digestive epithelium healthy, and that the lack of activation of AHR by bacterial tryptophan metabolites is associated with an aggravation of inflammatory bowel diseases [150]. Similarly, defective activation of AHR in the digestive tract plays a role in the development of metabolic syndrome [151], in gluten intolerance [152] and in liver alcohol toxicity [153].

Thus, if tryptophan-derived uremic toxins are dual in their activity, this is also the case for their main receptor AHR, which can have both beneficial and deleterious effects (Table 2), depending on the cell type, the ligand [154] and the time in the life of the organism [147]. This duality of action justifies understanding the pathways activated by AHR signaling to specifically block the deleterious pathways while maintaining the beneficial ones, mainly in cardiovascular disease, the main complication of CKD [155]. In the same way as for uremic toxins, AHR could be the Pharmakon of chronic kidney disease, at the same time being a cure and a poison [156].

## 4. Why Did the Body Continue Producing Toxic Tryptophan Metabolites throughout Evolution?

An intriguing question is why the generation of the noxious uremic toxins has persisted despite control mechanisms such as natural selection, reproductive isolation, and developmental plasticity. Of note, in this process, humans are not an isolated entity, but closely interact with the intestinal microbiome that generates a large array of uremic toxins or their precursors [157]. Due to developmental symbiosis, symbionts can impact phenotypic adaptation that subsequently may lead to genotypic accommodation [158]. Whatever the mechanism, the metabolic cascade leading to uremic toxins has not been switched off, nor has this been prevented by adapting gut microbial composition or function during evolution.

In this context, it is important to note that many of the ~600 genes that have been identified as potentially critical to the remote sensing and signaling network are found in gene families that have high evolutionary conservation [112]. Indeed, SLC22 transporters—the family in which OAT1 (SLC22A6) and OAT3 (SLC22A8) are found—are conserved in lower organisms and play an essential role in protecting fruit flies from oxidative injury [113]. Thus, it is conceivable that if selection tended to preserve the functioning of the remote sensing and signaling network, a key aspect of that functioning may be to optimize interorgan and inter-organismal small molecule communication in normal and perturbed states. The molecules necessary for optimal remote sensing and signaling include key metabolites, signaling molecules and antioxidants. Although some of these molecules also happen to be uremic toxins or other uremic molecules of the sorts already discussed, they and/or their biosynthetic pathways might be expected to be conserved throughout evolution.

**Figure 5:**
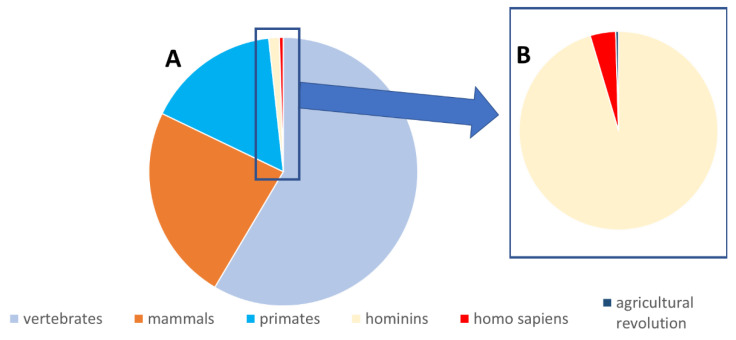
Evolutionary time scale of the main elements at play in uremic toxin generation on a 24 h scale. (**A**) Evolution starting with the vertebrates up to now; (**B**) enlargement of the boxed section in (**A**), starting with the hominins until now. If the vertebrates appear at time 0, homo sapiens appear only during the last minutes, and the agricultural revolution is only a fraction of the homo sapiens period. Whereas sulfotransferases appear long before the animals and the intestinal microbiome appears with the invertebrates (i.e., for both before this scale starts), the current conditions leading to an epidemic propensity of CKD appear only very late, with the agricultural revolution. However, this period is long enough to cover several hundreds of generations. The red section in panel (**A**) and the blue section in panel (**B**) are enlarged out of proportion to the other sections to allow visibility.

In addition, one must consider the possibility that the changes leading to uremic toxin generation came relatively late on the evolutionary timescale, so that there was not enough time for biologic correction. However, sulfotransferases, which are responsible for the conjugation of endo- and xenobiotics [159] including the sulfation of indoxyl to indoxyl sulfate, are present in mammals that made a much earlier evolutionary appearance than men [159,160], and even in more primitive organisms [161]. Thus, sulfotransferase has a long ancestral history originating many years before homo sapiens (Figure 5A).

Furthermore, symbionts, which include the intestinal microbiota, are ubiquitous across animals and plants and are in many cases essential for the functionality of the host [158]. The intestinal microbiome has been shown to be relatively consistent even across related species in conditions where dietary habits were similar [162], e.g., showing close similarities between non-human primates, Neanderthal men and hunter-gatherers [163], conforming to the thesis that symbiont microbiomes are relatively resilient [164]. The intestinal microbiome is also flexible depending on environment and lifestyle [165]. Additionally, CKD has an environmental impact [166,167,168], although the gut metabolome of hemodialysis patients also shows striking similarities with that of their household contacts without CKD [166], and the progression of CKD up to the stage of kidney failure treated by dialysis has no quantitative impact on the intestinal generation of toxins and their precursors [44]. Thus, also the intestinal microbiome processes generating uremic toxins seem to have been relatively well preserved over time.

Obviously, the risk of developing kidney disease itself, as we know it today [2], came latest of all. Although kidney disease must have existed since kidneys became functionally active, the circumstances promoting the current epidemical propensity were probably generated essentially with the agricultural revolution some 12,000 years ago, due to the transition to a more sedentary lifestyle [169,170,171] (Figure 5B), although the current exponential growth probably only started when obesity as a risk factor for diabetes overshadowed malnutrition as a nutritional problem [169,172]. However, such a period, although proportionally short compared to the other mechanisms at play in uremic toxin generation, still amounts to several generations. Given that adaptive variation due to environmental changes can occur relatively quickly [173,174,175], it might thus be assumed that, even when accounting for the history of CKD, timing may be less likely an explanation for the evolutionary persistence of uremic toxin production, and that other factors are more important.

It might also be that the involved mechanisms have other functions that are useful or even indispensable for preserving the hosting species, its symbionts or, according to the holobiont concept, the interaction between both. Conjugation mechanisms such as sulfation and glucuronidation are nonspecific and play a role not only in the generation of uremic toxins but also in the detoxification of xenobiotics, such as phenolic food components [159]. The gut microbiome is essential for the development of an efficacious immune system [176,177], gastro-intestinal motility [178], platelet function [178], brain functioning [179,180] and psychological health [179,180,181], and not only produces uremic toxins but also many beneficial compounds, as particularly exemplified by the tryptophan metabolites. In addition, some of these metabolites (indole-3-acetic acid, indole-3-propionic acid), which in plant physiology act as growth hormones [182,183] (auxins), may also stabilize the intestinal microbiome and preserve it against pathogen intrusion [56,184]. The AHR, which is activated by several toxic tryptophan metabolites resulting in a thrombogenic response [124,126], but also by non-toxic tryptophan metabolites [68], is also essential for detoxification [127,129,130] and immune regulation [127,128]. Thus, the metabolic system generating the tryptophan metabolites may be too valuable to be discarded, especially since the production of uremic toxins does not matter to most people, in whom kidney function is normal or close to normal. In addition, uremic retention solutes are probably only toxic at increased concentrations, causing no or only little harm for a host in good health. Finally, in most people developing kidney disease, this problem only occurs at a relatively advanced age, thus precluding reproductive isolation [158]. This does not, however, exclude the possibly of the occurrence of other, possibly more limited, phenotypic changes such as partial resistance to biological effects.

## 5. Is the Example of Tryptophan Metabolites Representative for Uremic Retention at Large?

Tryptophan metabolism may be unrepresentative for other uremic toxin families or mechanisms, but it may also be that other uremic patho-mechanisms are counterbalanced as well. This is biologically plausible, as it provides a fine-tuning mechanism preventing amplification or overly damaging effects.

For example, pro-inflammatory peptides including cytokines, which are retained and generated in uremia [185], are counteracted by their circulating receptors or anti-inflammatory cytokines (Table 3). The anorexigen desacyl-ghrelin counterbalances the orexigen ghrelin [186]. Despite many arguments in favor of a negative cardio-vascular impact [187], trimethyl amine-N-oxide is also known as a protein stabilizer [188]. The toxicity of urea [189] may be neutralized by another group of uremic retention solutes, the methylamines [190]. Uric acid can act both as a pro-oxidant and an antioxidant [191,192]. Finally, among the larger peptides (middle molecules) that are retained in uremia, alongside compounds with toxic potential, some, such as adrenomedullin, atrial natriuretic peptide, glomerulopressin and visfatin, have a positive rather than a negative biological impact [6,185]. Admittedly, the toxic effect of uremic retention solutes is supported by more scientific evidence than the potentially positive effects, but these alternative routes may have been explored insufficiently and the potential for publication bias cannot be ruled out.

Some may argue that this reasoning does not apply for many other groups of intestinally generated uremic toxins, such as the cresols and the phenols, which are largely classified as toxic [6,193]. However, to the best of our knowledge, very few metabolites of these pathways have been fully evaluated from the perspective of potentially beneficial effects. It is possible that if research were to be extended to a broader array of metabolites of the mother compounds in the same way as for tryptophan, this would allow for detecting some yet insufficiently understood substances. Interestingly, different steric variants of cresyl glucuronide had an opposite impact on human embryonic kidney cells [194]; whereas m- and p-cresyl glucuronide were toxic, o-cresyl glucuronide slightly stimulated cell growth [194]. Dihydroxy phenyl propionic acid has been characterized as an anti-inflammatory agent [195]. Phenyl aldehyde had a damaging effect on *Candida albicans*.

Whatever the pathophysiologic impact of a broader array of compounds than those we usually consider, the same argumentation as the one held above—in other words, that the mechanisms leading to uremic toxin production were not switched off because the preservation of the species was not (sufficiently) endangered—still applies.

## 6. Summary and Future Outlook

The message conveyed in this publication is that solutes emanating from the same origin as uremic toxins may be beneficial or even essential for body functioning. The RSST is a useful construct for thinking about small molecule communication between organs and organisms to preserve normal physiology and counteract pathophysiological states. Likewise, AHR, the main receptor of the tryptophan metabolites, also has been linked both to harms and benefits. In addition, as for other poisonous compounds, uremic poisons might also, under certain conditions and at certain concentrations, have positive effects, as exemplified in this text for the solutes that were labelled as ambivalent, but also for an accepted toxin such as indoxyl sulfate.

As this text proposes a hypothesis, it is by definition provocative and formulates a number of as yet not consolidated viewpoints, and parts of what is formulated may be refuted in the future when new knowledge appears. On the other hand, this text was formulated only after ample literature search and involves elements related to several different biological pathways and systems. Additionally, this publication essentially focuses on tryptophan metabolism, as acknowledged in Section 5, although also a limited number of other examples that are not related to tryptophan are illustrated. However, the focus on tryptophan is unavoidable in view of the scarcity of data on other pathways and factors.

More important than the question of why the production of uremic toxins has been preserved is to consider the consequences of this functional dualism for further analysis and treatment of uremic toxicity (Table 4). The baby should not be thrown away with the bath water. Specifically for tryptophan metabolites, a complete mapping of the evolution of concentrations of all involved compounds throughout the progression of kidney disease should offer a clear insight in the basic condition without therapeutic intervention. This infers that such analysis would not only include the usual suspects (presumed toxins), but also beneficial and ambiguous compounds, as depicted above, to assess in what direction their concentrations change as kidney dysfunction progresses. Additionally, research on biological effects in CKD should be extended to a broader array of metabolites. This implies that any study assessing treatment options to decrease uremic toxin concentration should consider the impact on the complete metabolite balance—with special attention to the molecules with a favorable effect, to clarify whether or not beneficial compounds are removed or affected in the same way as the toxins. Maybe a simultaneous removal of compounds with positive and negative effects explains the deceiving results of several controlled studies comparing high efficacy dialysis strategies such as on-line hemodiafiltration compared to standard treatment [196,197].

In the context of the RSST, one can envision a homeostatic system attempting to restore itself via small molecule interorgan (and inter-organismal) communication in the setting of CKD and between dialysis sessions. Dialytic removal of some small molecules that are beneficial to this endogenous restoration effort (remote sensing and signaling) may partly defeat the purpose, especially if these beneficial molecules tend to decrease inflammation, cardiovascular and endothelial damage, or fibrosis. It follows, then, that therapeutic options which maintain or restore the balance between positive and negative effects are to be preferred over those aimed only at eliminating toxins but at the expense of worsening the imbalance. In this sense, pre-, pro-, syn- or postbiotics [198] or strategies preserving kidney function may be more physiological than removal strategies; in terms of the RSST, such strategies may be more prone to promoting beneficial inter-organismal (e.g., gut microbe–host) communication via small molecules, and this question should be answered. Finally, it is desirable that scientific journals actively solicit reports of suitable quality on the neutral or beneficial effects of apparent uremic toxins, even if these results conflict with existing knowledge. Likewise, it is the responsibility of researchers not to shelve such atypical results.

In addition, a more extended metabolic review and study of the metabolites of other intestinally generated precursors (cresol, phenol, hippurates) might also reveal as yet insufficiently explored perspectives. In particular, the dualism of the metabolites of other amino acids than tryptophan (e.g., tyrosine and phenylalanine for p-cresol) should be explored with an open mind for counterbalancing elements dampening toxic effects. For all metabolic pathways, determination of concentration thresholds determining toxicity vs. benefit might also be relevant. As our understanding of the remote sensing and signaling network becomes more detailed, it may also be possible to assess the impact of “non-normal” levels of these uremic small molecules on communication pathways between cells, organs, and organisms. The results might in turn lead to reconsidering therapeutic options, e.g., by stimulating certain specific pathways and inhibiting others, or by trying not only to affect intestinal generation but also absorption by intestinal epithelial cells.

Another intriguing but unanswered question is whether a relative increase in uremic toxins with normal kidney function has a clinical impact or not. Although observational, a study by Glorieux et al. including the entire spectrum of CKD stages whereby the link of free p-cresyl sulfate to cardio-vascular disease remained significant after adjustment for several risk factors, including estimated glomerular filtration rate [199], may point in this direction, but this should be further analyzed.

## Figures and Tables

**Table 1:** Metabolites of the tryptophan pathway with their chemical structures and the involved enzymes.

Metabolite	Structure Formula	Enzymes Involved
Tryptophan	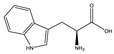	
**Indole pathway**		
Indole	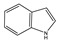	Tryptophanase
Indoxyl sulfate	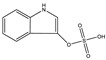	Sulfotransferase/CYP2E1
Indoxyl glucuronide	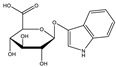	Glucuronyltransferase
Indole-3-propionic acid	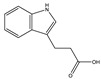	Tryptophanase (?)
Indole-3-acetic acid	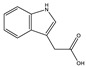	Aldehyde dehydrogenase (NAD+)/indole-3-acetaldehyde oxidase/IL4I1
Indole-3-(carbox)aldehyde	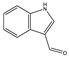	Aromatic-L-amino-acid/L-tryptophan decarboxylase
**Kynurenine pathway**		
L-Kynurenine	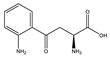	Tryptophan 2,3-dioxygenase or indoleamine 2,3-dioxygenase/arylformamidase
Kynurenic acid	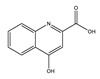	Kynurenine-oxoglutarate transaminase/cysteine-S-conjugate beta-lyase/glutamine-phenylpyruvate transaminase
Anthranilic acid	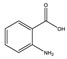	Kynureninase
Quinolinic acid	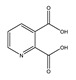	Anthranilate 3-monooxygenase (FAD)/4-hydroxyphenylacetate 3-monooxygenase/3-hydroxyanthranilate 3,4-dioxygenase
Nicotinic acid (niacin) (*Nicotinate and Nicotinamide metabolism*)	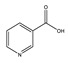	Nicotinate-nucleotide pyrophosphorylase (carboxylating)/nicotinate phosphoribosyltransferase
Nicotinamide (*Nicotinate and Nicotinamide metabolism*)	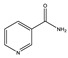	Nicotinate-nucleotide adenylyltransferase/nicotinamide adenine dinucleotide (NAD)+ synthase/NAD+ diphosphatase/5′-nucleotidase
N1-Methyl-2-pyridone-5-carboxamide (*Nicotinate and Nicotinamide metabolism*)	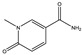	Nicotinamide methyl transferase/aldehyde oxidase
**Serotonin Pathway**		
Serotonin	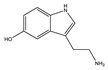	Tryptophan 5-monooxygenase/aromatic-L-amino-acid/L-tryptophan decarboxylase
Melatonin	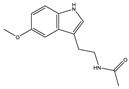	Arylalkylamine N-acetyltransferase/acetylserotonin O-methyltransferase

IL4I1: interleukin 4-induced-1 (IL4I1); CYP2E1: cytochrome P450 family 2 subfamily E member 1.

**Table 2:** Duality in the response to activation of aryl hydrocarbon receptor.

Positive	Negative
Detoxification	Pro-inflammatory effect
Preservation vascular structure	Chloracne
Closure ductus venosus (post-partum)	Lymphoma
Normalization blood pressure	Malformative syndrome
Preservation liver function	Carcinogenicity
Prevention fibrosis	Prothrombotic effect
Prolongation healthy life	Vascular toxicity
Forestalls ageing	Neurotoxicity
Preservation digestive epithelial function	Auto-immune diseases
Prevention inflammatory bowel disease	Zika infection
Prevention metabolic syndrome	
Prevention gluten enteropathy	
Prevention alcohol toxicity	

**Table 3:** Opposite mechanisms in families of peptidic uremic retention compounds.

Toxic	Neutral or Non-Toxic
Complement factor D	Complement factor Ba
Interleukin-1β	Interleukin-1 receptor antagonist
Tumor necrosis factor-α	Soluble tumor necrosis factor receptor
Interleukin-6	Interleukin-10
Cholecystokinin	Ghrelin
Desacyl Ghrelin	Ghrelin
Leptin	Orexin A
Peptide YY	Neuropeptide Y

**Table 4:** Recommendations for future consideration.

Tryptophan metabolites
Provide a complete mapping of the evolution of compounds with positive and negative biological impacts throughout all CKD stages.
Extend biological research to a broader array of compounds than the usual suspects
Assess the effect of therapeutic strategies on molecules with as well positive as negative biological effects.
Consider choosing therapies that maintain or restore the balance between components with positive and negative impact, rather than removing toxins as well as beneficial compounds.
Promote the publication of suitable studies showing atypical results of uremic toxin actions.
Researchers should not shelve uremic toxin research with atypical results.
**Other than tryptophan metabolites**
Develop extensive reviews and studies on a broader array of metabolites than the ones frequently considered now.
Based on this knowledge, extend the analysis and development of therapeutic options.

## Data Availability

Not applicable.
